# Characterization and Antifungal Activity of Lemongrass Essential Oil-Loaded Nanoemulsion Stabilized by Carboxylated Cellulose Nanofibrils and Surfactant

**DOI:** 10.3390/polym15193946

**Published:** 2023-09-29

**Authors:** Lingling Liu, Kaleb D. Fisher, Mason A. Friest, Gina Gerard

**Affiliations:** 1 Department of Agricultural and Biosystems Engineering, Iowa State University, Ames, IA 50010, USA; 2 Roy J. Carver Department of Biochemistry, Biophysics and Molecular Biology, Iowa State University, Ames, IA 50010, USA; 3 Department of Mechanical Engineering, Iowa State University, Ames, IA 50010, USA; mafriest@iastate.edu; 4 Department of Food Science and Human Nutrition, Iowa State University, Ames, IA 50010, USA

**Keywords:** corn stover, TEMPO-CNF, thermodynamic stability, *Aspergillus flavus*

## Abstract

Nanocellulose is an emerging green, biodegradable and biocompatible nanomaterial with negligible toxicities. In this study, a carboxylated nanocellulose (i.e., 2,2,6,6-tetramethylpiperidin-1-oxyl (TEMPO)-oxidized cellulose nanofibril (TEMPO-CNF)) was prepared from corn stover and characterized by X-ray diffraction (XRD), Fourier Transform Infrared Spectroscopy (FTIR) and differential scanning calorimetry (DSC)/thermogravimetric analysis (TGA). Corn stover-derived TEMPO-CNF was explored as an emulsion co-stabilizer together with Tween 80 for lemongrass essential oil-loaded emulsions. Droplet size, phase behavior and thermodynamic stability of oil-in-water emulsions stabilized by Tween 80 and TEMPO-CNF were investigated. The optimal nanoemulsion stabilized by this binary stabilizer could achieve a mean particle size of 19 nm, and it did not form any phase separation against centrifugal forces, freeze–thaw cycles and at least 30 days of room temperature storage. The nanoencapsulated essential oil had better inhibition activity against the mycelial growth of *Aspergillus flavus* than pure essential oil. Results from this study demonstrate the potential of using agricultural byproduct-derived nanomaterial as nanoemulsion stabilizers for essential oils with good emulsion thermodynamic stability as well as enhanced antifungal activities.

## 1. Introduction

Pesticides, including herbicides, insecticides and fungicides, have been widely used in modern agriculture to protect plants, especially crops. However, pesticides, especially chemical pesticides, cause a number of problems, including the emergence of drug-resistant pests and the runoff of the remaining pesticides into the groundwater. In addition, the use of chemical pesticides can also cause chemical accumulation in the food chain, resulting in health concerns. Biopesticides are materials with pesticidal properties that originate from plants, animals and microorganisms. Biopesticides, which are bio-derived from organisms, have been shown to be promising since they are safer and more environmentally friendly compared to their chemical counterparts. Currently, biopesticides only comprise 5% of the total crop protection market worldwide, with a value of ~3 billion USD. Although the use of biopesticides is increasing by ~10% every year, the global market for biopesticides needs to increase in order to substitute chemical pesticides in the long run [1]. Therefore, there is an urgent need to investigate novel and effective ways to improve the efficiency of biopesticides.

Plant essential oil is a type of biopesticide that is originated from plants and has been shown to be effective against plant disease pathogens and pests [2,3]. For instance, cinnamon bark (*Cinnamomum zeylanicum*) and tea tree (*Melaleuca alternifolia*) essential oils showed inhibitory effects against bacteria, fungi, yeast and viruses [4]. In addition, essential oils of *Cinnamomum zeylanicum* (cinnamon), *Origanum vulgare* (origanum), *Mentha piperita* (peppermint), *Ocimum basilicum* (basil), *Thymus vulgaris* (thyme) and *Syzygium aromaticum* (clove) showed antifungal activity against *Aspergillus flavus* in maize [5]. Essential oils, including cinnamon (*Cinnamomum zeylanicum*), clove (*Eugenia caryophyllata*), lemongrass (*Cymbopogon flexuosus*) and thyme (*Thymus vulgaris*), are generally recognized as safe (GRAS) materials. However, essential oils suffer from fast release and low stability [6,7,8,9]. Enhancing the stability of essential oils against environmental stresses are one of the main factors to be considered when utilizing essential oils for biocontrol applications [6]. Therefore, finding innovative ways to improve the stability of essential oils would be beneficial for improving their biopesticidal efficacy.

Among these, the nano-encapsulation approach has been shown to be very effective at enhancing the stability as well as improving the efficacy of biopesticides [10,11]. Nano-encapsulated biopesticides could exert higher pesticidal activity than microencapsulated ones due to increased local concentration of biopesticides and enhanced passive cellular absorption [12]. Research studies show that nano-formulations can improve the efficacy of pesticides by up to 10-fold, with a median gain in efficacy of ~20% compared to conventional analogs [13]. Nanoencapsulation systems often protect biopesticides from adverse conditions and modulate their release.

Among all nanomaterials, nanocellulose is an organic nanomaterial that is nontoxic, biodegradable and biocompatible. Nanocellulose with at least one dimension within 100 nm can be prepared from cellulosic materials with chemical treatments (e.g., acid hydrolysis) [14], mechanical shearing [15,16], enzymatic treatments [17] and biosynthesis (i.e., bacteria nanocellulose) [18]. Research studies show that nanocellulose can be used as an emulsion stabilizer [19,20].

Nanocellulose can be prepared from agricultural and food byproducts like corn stover and soybean residues, which have a high percentage of cellulose and are often underutilized [21,22]. In our previous study [23], a carboxylated nanocellulose (TEMPO-CNF) was prepared from corn stover, and it could work as a Pickering emulsion stabilizer for lemongrass essential oil (EO)-loaded emulsion. The emulsion stabilized by a binary emulsion stabilizer (i.e., TEMPO-CNF and Tween 80) was physically stable against centrifugal forces and freeze–thaw cycles, indicating that lemongrass EO can be well encapsulated by this binary emulsion stabilizer [23]. In this study, we intended to further characterize the chemical composition, crystal structure and thermal properties of corn stover-derived TEMPO-CNF, as well as investigate the properties of EO-loaded emulsion and its antifungal effect against *Aspergillus flavus*. *Aspergillus flavus* is a fungus that can cause ear rots in maize and is associated with the production of aflatoxins that cause serious safety concerns [24]. We hypothesized that lemongrass EO encapsulated by TEMPO-CNF and Tween 80 had higher antimicrobial activity than pure EO. Results from this study demonstrate the potential application of corn byproduct derived nanocellulose as an encapsulation agent for biopesticides.

## 2. Materials and Methods

### 2.1. Materials

Lemongrass essential oil (*Cymbopogon Schoenanthus* Oil, reagent grade, 100%) was purchased from Spectrum Chemical MFG Corp (New Brunswick, NJ, USA). Tween 80 (P1754) was purchased from Sigma-Aldrich, Inc. (St. Louis, MO, USA). *Aspergillus flavus* Link 200026 NRRL 3357 strains purchased from ATCC (Manassas, VA, USA) were used. All other chemicals and supplies were purchased from VWR (Radnor, PA, USA) or Fisher Scientific (Pittsburgh, PA, USA). Corn stover-derived TEMPO-CNF was prepared according to our previous publication [23]. Specifically, corn stover was ground to a size below 1/8 inch, followed by washing to remove impurities. Then, corn stover was bleached to remove lignin, followed by alkaline treatment to remove hemicellulose. Lastly, corn stover went through TEMPO oxidation and sonication to achieve TEMPO-CNF. Corn stover-derived TEMPO-CNF had a length of 353 nm, width of 4 nm, surface charge of 1.48 ± 0.09 mmol/g COO^−^ and zeta potential of −65 ± 3 mV [23]. The as-prepared TEMPO-CNF gel had a solid content of ~3.8 wt% [23].

### 2.2. Characterization of TEMPO-CNF

Corn stover-derived TEMPO-CNF was freeze-dried (Labconco, Kansas City, MO, USA), then characterized by X-ray diffraction (XRD), Attenuated Total Reflectance Fourier Transform Infrared Spectroscopy (ATR-FTIR) and differential scanning calorimetry (DSC)/thermogravimetric analysis (TGA). XRD measurement was performed using an X-ray diffractometer (Ultima IV, Rigaku Americas Corporation, Woodlands, TX, USA) operated at a voltage of 40 kV and a current of 44 mA. The sample was scanned with a scanning rate of 2°/min within the range of 10° to 50° for 30 min at room temperature. Data were acquired, and the crystallinity of the samples was calculated by the Segal method (Segal, 1959), as shown in Equation (1a).

(1a) $$CrI\;(\%)=\frac{I_{002}-I_{AM}}{I_{002}}\times 100$$

In Equation (1a), CrI refers to crystallinity index, $I_{002}\;$ refers to the maximum intensity of the (002) lattice diffraction (at 2θ of 22~24°), $I_{AM}\;$ refers to the minimum intensity of diffraction between (002) and (110) planes (at 2θ around 18°). $I_{002}\;$ represents both amorphous and crystalline regions, while $I_{AM}$ only represents an amorphous region [25].

The as-prepared TEMPO-CNF was also measured using ATR-FTIR spectroscopy (Bruker Tensor 37, Bruker Scientific LLC, Billerica, MA, USA). Specifically, 1% sample and 99% spectral grade KBr were mixed and pressed into a pellet for the measurement in the region of 600~4500 cm^−1^ at a resolution of 4 cm^−1^, averaging 32 scans per sample. Background spectra were also collected by using KBr only, and baseline correction was made by subtracting the background spectra.

DSC/TGA characterization was performed by using the STA 449 F1 Jupiter thermal analyzer (NETZSCH Instruments North America, LLC, Burlington, MA, USA). Thermal stability of sample was monitored with a temperature range of 40~800 °C under nitrogen with a flow rate of 10 mL/min and a constant heating rate of 10 °C/min. Derivative thermogravimetric (DTG) curve was generated to show the rate of mass change of the materials with respect to temperature against temperature changes.

### 2.3. Preparation of Emulsion Stabilized by TEMPO-CNF and Tween 80

Emulsions were prepared by using both TEMPO-CNF and Tween 80 as stabilizers. The EO-loaded emulsion contained certain concentrations of lemongrass EO (2.5 wt%, 5 wt%, 10 wt% and 20 wt%), certain concentrations of Tween 80 (EO/Tween 80 ratio 1:3, 1:1 and 3:1), and certain concentrations of nanocellulose (0 and 0.3 wt%). Emulsions were prepared by mixing aqueous phase (containing nanocellulose and water) and oil phase (Tween 80 and EO) together with magnetic stirring, followed by sonication with a 500 W ultrasonicator (Fisher Scientific, Hampton, NH, USA) at 100% amplitude for 5 min in an ice bath.

### 2.4. Thermodynamic Stability Testing of Emulsions

Several tests, including centrifugation, freeze–thaw and room temperature storage tests of emulsion samples, were performed in order to assess their thermodynamic stability. Freshly prepared emulsion samples were centrifuged at 10,000 rpm (Eppendorf 5418, Enfield, CT, USA) for 15 min at 23 °C and observed for phase separation (if any). Emulsion samples were also observed for phase separation (if any) after 2 cycles of freeze–thaw process, with each cycle stored at −20 °C for 48 h and 25 °C for 48 h. Emulsion samples were also stored at room temperature for 30 days (away from light) and observed for phase separation.

### 2.5. Determination of Emulsion Particle Size and Surface Charge (Zeta Potential)

The particle size and zeta potential of the emulsion stabilized by TEMPO-CNF and Tween 80 (labeled as EO + T80 + TCNF) were determined right after emulsion preparation by using a Zetasizer Nano ZS instrument (Malvern Instruments Ltd., Worcestershire, UK). EO + T80 + TCNF refers to the emulsion formulated with 2.5% EO, 7.5% Tween 80 and 0.3% TEMPO-CNF. Emulsion stabilized by Tween 80 only (labeled as EO + T80) was also prepared and determined for its particle size and surface charge. As a comparison, an emulsion sample without the stabilizer Tween 80 or TEMPO-CNF (labeled as EO + W) was also prepared by the mixture of EO and water. All the emulsion samples were prepared as described in Section 2.3.

The mean hydrodynamic diameter (z-average), polydispersity index and zeta potential (ζ-potential) of the emulsions were measured using a Zetasizer (Malvern Zetasizer Nano ZS, Malvern Panalytical Ltd., Malvern, UK) based on the dynamic light scattering technique. All tests were performed in triplicate at 23 °C. Material and dispersant refractive index are 1.50 and 1.33, respectively. Samples were diluted with deionized water (1:100) before measurement.

### 2.6. Antifungal Activity Assay

#### 2.6.1. Mycelial Growth Inhibition Assay

The effectiveness of nanoencapsulated essential oil was determined by the *in vitro* inhibition of mycelial growth using the ‘poison food’ technique [26] with some modifications. Four sample groups were tested, including EO, EO + W, EO + T80 and EO + T80 + TCNF. The emulsion samples were prepared as described in Section 2.5. Pure EO was studied as a comparison. Potato dextrose agar (PDA) containing 0.1% (*v*/*v*) Tween 80 was prepared and used as the media. Once media was autoclaved, it was placed in a water bath and maintained at a temperature of 55 °C. Then certain volumes of EO or parent emulsion were added to a cool, sterile molten PDA medium to achieve certain EO concentrations. *Aspergillus flavus* (ATCC^®^ 200026™) was inoculated with a volume of 10 μL (10^5^ conidia/mL) on the dried PDA plates. Plates were incubated at 25 °C, and the diameter of mycelium growth was recorded over 14 days. Four replicates (with 2 plates per replicate) were performed for each sample. *A. flavus* conidia was prepared by scraping off the agar surface of 14-day-old culture and suspended in sterile water, followed by counting using a hemocytometer. The fungicidal activity of the samples was determined based on the percentage of mycelial growth inhibition (MGI) using the equation below.

(1b) $$MGI\left(\%\right)=\frac{d_{c}-d_{t}}{d_{c}}\times 100$$

where $d_{c}$ and $d_{t}$ refer to the diameter of mycelial growth in control and treated plates, respectively.

In order to assess the effect of different emulsion preparation treatments on the antifungal activity of encapsulated lemongrass essential oil, several treatment conditions were compared, including stirring, sonication, homogenization and homogenization followed by sonication. All these treatment conditions were common in the preparation of encapsulated essential oils. The sample formulation EO + T80 + TCNF was used. For sonication treatment, the sample was sonicated as described above. For stirring treatment, the components EO, Tween 80 and TEMPO-CNF were stirred well without further treatment. The homogenization treatment was performed by subjecting the emulsion to a homogenizer (Biospect Products, Bartlesville, OK, USA) at 10,000 rpm for 2 min. Homogenization followed by sonication treatment was carried out by subjecting the emulsion to homogenization first and subsequently treated by sonication. The mycelial growth inhibition assay for these samples was performed as above.

#### 2.6.2. Conidial Germination Inhibition Assay

To determine the effectiveness of the nanoencapsulated essential oil against spore germination of *Aspergillus flavus*, a conidial germination inhibition assay (so-called ‘cavity slide’ technique) was used [26] with modifications. Sterile potato dextrose broth (PDB) with 1% Tween 80 was prepared, then certain volumes of EO or parent emulsion were added to achieve certain EO concentrations. Then, 40 µL of the PDB containing EO or emulsion was added on a cavity slide containing 10 µL of spore suspension (10^5^ conidia/mL). Cavity slide containing spore suspension and PDB with 1% Tween 80 was also conducted as a control. The inoculated slides were placed inside a petri plate with a moist filter paper at the bottom and incubated at 25 °C for 24 h. Then, 10 µL of 2% sodium azide was added to stop the germination. Conidial germination was then evaluated by using an optical microscope. A conidium is considered germinated if the length of the germ tube is at least twice the conidium diameter. The percent inhibition of germination (IG) is calculated using the equation below. Conidial viability was calculated by the percentage of the germinated conidia among ~150 counted conidia. Three replicates were performed for each sample.

(2) $$IG\left(\%\right)=\frac{G_{c}-G_{t}}{G_{c}}\times 100$$

where $G_{c}$ and $G_{t}$ refer to the conidial viability of germinated conidia in control and treated slides, respectively.

### 2.7. Statistical Analysis

Significance analysis (*p* < 0.05) was performed by using Duncan’s multiple comparison tests with SAS/STAT software (SAS Institute Inc., Cary, NC, USA).

## 3. Results and Discussion

### 3.1. XRD Characterization

**Figure 1:**
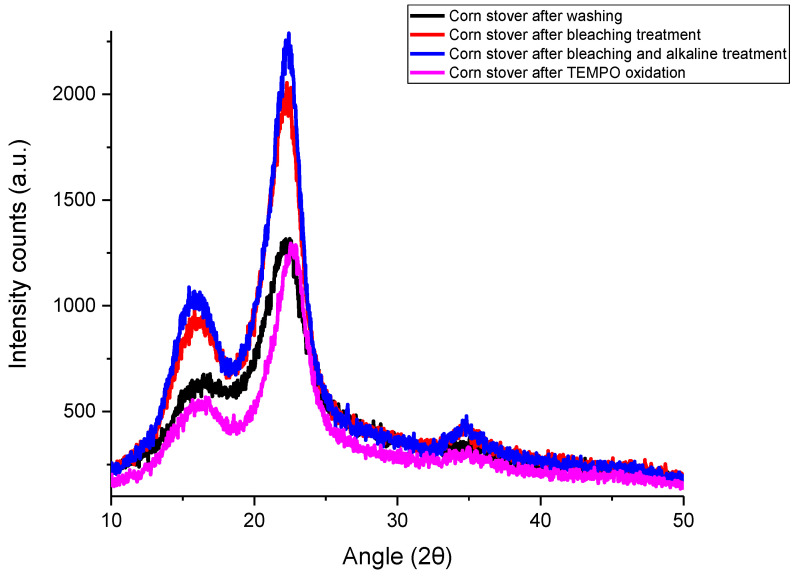
X-ray diffraction (XRD) patterns of corn stover samples after each treatment.

Figure 1 shows the XRD patterns of corn stover samples after each treatment. Corn stover after washing had three reflection peaks at 2θ = 16.5°, 22.2° and 35.1°. These peaks had also been reported by Costa, Assis, Gomes, da Silva, Fonsêca and Druzian [21] for corn stover samples, and these peaks are characteristics of cellulose I crystals. The peaks at 2θ = 16.5° and 22.2° correspond to (110) and (002) crystallographic planes, respectively [21,27]. The peak at 35.1° corresponds to (004) planes [27]. After the removal of lignin and hemicellulose by bleaching and alkaline treatment, narrower crystalline peaks were observed and the intensities of peaks were more defined. Similar phenomena were observed for corn stover samples [21] and corn husks [27].

According to Table 1, the calculated crystallinity index of corn stover after washing was 57.07%, while the crystallinity index increased to 67.20% after bleaching treatment. With further alkaline treatment, the crystallinity index increased to 69.95%. Not much change in crystallinity index was observed after TEMPO oxidation treatment, as TEMPO-CNF has a crystallinity index of 69.50%.

The crystallinity index of corn stover after washing (CS) in this study (57.07%) is similar to that reported for corncob residues (~61% crystallinity) [28] but higher than that reported for water-soaked corn husk (36.61% crystallinity) [27] and corn stover (~25% crystallinity) [21]. After bleaching and alkaline treatment, lignin and hemicellulose were removed, respectively, which enhanced the crystallinity index of corn stover samples. Similar trends were observed by Kampeerapappun [27], of which the crystallinity index increased ~13% and 5% after bleaching and alkaline treatment, respectively. In this study, a similar crystallinity index was observed between CS-BAT and TEMPO-CNF. Similarly, it was shown that the TEMPO oxidation process results in nearly no change in the crystallinity value and crystal size of cellulose cotton linter [29] or wood cellulose [30]. But, there are also some studies reported that lower values of crystallinity were observed after TEMPO oxidation and fibrillation treatment [31,32,33]. The crystallinity index of TEMPO-CNF in this study (69.5%) is similar to that reported for freeze-dried TEMPO-CNF derived from rice straw (66.3~74.5% crystallinity) [34], TEMPO-CNF from *Picea abies* spruce sulfite pulp (68% crystallinity) [35] and TEMPO-CNF from the lower part of empty fruit bunches (73.69% crystallinity) [31]. But, the crystallinity index of TEMPO-CNF derived from corn stover (this study) is much lower than that of TEMPO-CNF derived from softwood bleached kraft pulp or microcrystalline cellulose which had crystallinity indices of 81% and 88%, respectively [36].

### 3.2. FTIR Characterization

**Figure 2:**
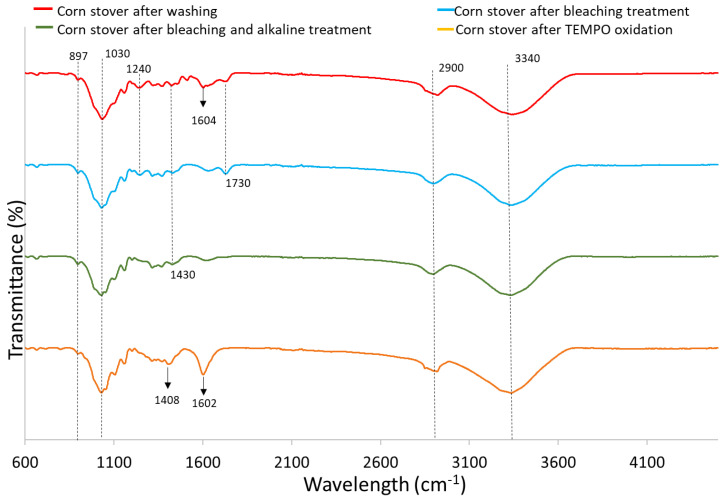
FTIR spectrum of corn stover samples after each treatment.

Figure 2 shows the FTIR spectrum of corn stover samples after each treatment. The peaks found between 890 and 1100 cm^−1^ have been reported to be associated with the C-H vibration and C-O stretch of cellulose [37]. Specifically, the small peak at 897 cm^−1^ represents the β-glycosidic linkage of cellulose [38]. The significant peaks displayed by all samples at around 1030 cm^−1^ indicate the C-O stretch bonds characteristic of esters, confirming the cellulose structure of samples. The peaks observed at around 1240 cm^−1^ for corn stover after washing (CS) and corn stover after bleaching treatment (CS-BT) denote the aryl group of lignin whereas it is not found for the alkaline treated sample and TEMPO-CNF, indicating the removal of lignin. The peak at 1430 cm^−1^ referring to symmetrical CH_2_ bending is present for CS, CS-BT, as well as corn stover after bleaching and alkaline treatment (CS-BAT) samples, but the peak shifted to 1408 cm^−1^ in the corn stover after TEMPO oxidation (TEMPO-CNF) sample indicating the formation of new hydrogen bonds [38].

CS exhibits a characteristic peak around 1604 cm^−1^ linked to C-C stretch aromatic bonds. But, there is a visible reduction in the intensity of this peak for CS-BT and CS-BAT, which is associated with the breakage of lignin side chains by the bleaching treatment [21,39]. The band at 1602 cm^−1^ for TEMPO-CNF was significantly stronger than the other samples due to antisymmetric COO^−^ (carboxyl) stretching. It was reported that the TEMPO-mediated oxidation process introduces carboxyl groups post-process [40]. The peaks found at 1730 cm^−1^ for CS and CS-BT correspond to C=O ester-linked acetyl and uronic acid groups in hemicelluloses or ester-linked carboxylic groups in the ferulic and p-coumaric acids found in lignin [21,28,39]. However, this peak is not present in CS-BAT and TEMPO-CNF due to the removal of hemicellulose and lignin by bleaching and alkaline treatments.

All samples tested exhibited a strong, broad peak at ~3340 cm^−1^ due to the O-H stretching vibration of hydroxyl groups in cellulose and hemicellulose. These groups are responsible for the moisture absorption capacity of biomass [40,41]. All samples tested show a peak around 2900 cm^−1^, which is attributed to the symmetric and asymmetric stretching vibrations of -CH, -CH_2_ and hydroxyl groups in cellulose, hemicellulose and lignin [38]. Similar peaks were observed in corn stover samples [42,43] and cellulose nanofibers from office waste paper [38].

### 3.3. DSC and TGA Characterization

**Figure 3:**
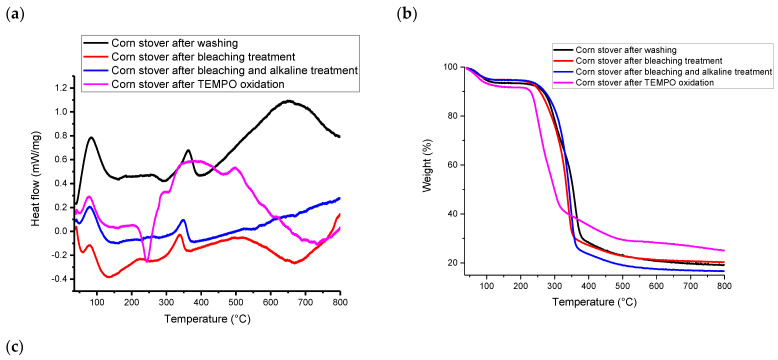

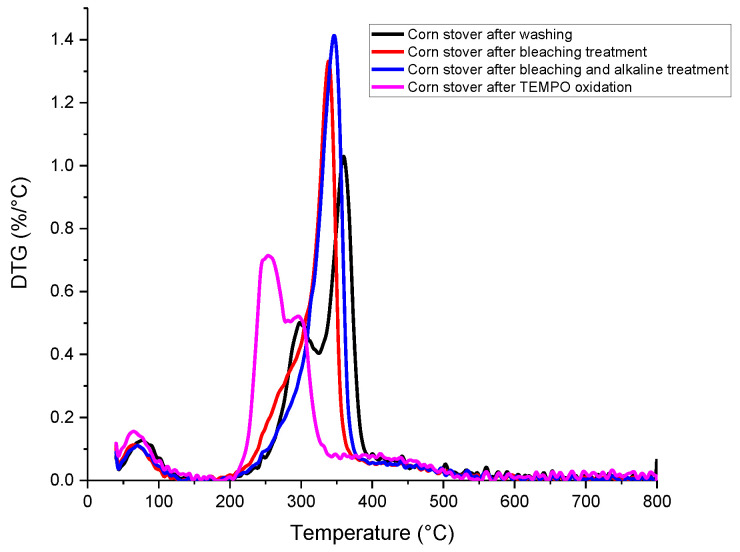
(**a**) DSC, (**b**) TGA and (**c**) DTG analysis of corn stover samples after each treatment.

According to Figure 3, all samples had an exothermal peak and weight loss at the temperature range of 25~160 °C due to the evaporation of absorbed moisture on the material surface, including inter-molecularly H-bonded water and chemisorbed water [21]. TEMPO-CNF underwent more weight loss at temperatures above 100 °C as compared to other samples. Specifically, the initial weight loss of TEMPO-CNF is 7.8 wt%, while it is 4.9~6.2 wt% for other samples. According to Table S1 (Supplementary Materials), the onset decomposition temperature of TEMPO-CNF was 235 °C, which is lower than that of other samples (246 °C~284 °C). Compared with other samples, the presence of carboxyl groups in TEMPO-CNF led to a decrease in its thermal degradation temperature. This is because the carboxyl moiety disturbs and breaks down the strong hydrogen bonds among cellulose fibrils. This finding is similar to that reported for TEMPO-oxidized hardwood bleached chemi-thermomechanical pulp [44] or TEMPO-oxidized kraft pulp [45].

According to Figure 3b,c, at temperatures above 200 °C, TEMPO-CNF showed two weight loss events, corresponding to two peaks in the DTG curve. Specifically, cellulosic materials with surface groups typically have two weight loss events, with the first weight loss event attributing to the surface charge group (i.e., the carboxyl group in this study) and the second weight loss event attributing to the hydroxyl group [46]. The weight loss pattern of corn stover-derived TEMPO-CNF in this study is similar to that of TEMPO-oxidized bagasse pulp [47] and TEMPO-oxidized rice straw cellulose [34]. In addition, according to Figure 3a, TEMPO-CNF has a much broader exothermal peak than other samples. Specifically, TEMPO-CNF had a broad exothermal peak at around 384 °C, while other corn stover samples had a narrower peak at 338~363 °C. The broad exothermal peak was also observed in TEMPO-oxidized rice straw cellulose [34], as well as cellulose and nanocellulose prepared from Betel nut fruit husk fibers [48].

The weight loss pattern of corn stover after washing (this study) is also similar to that reported by Costa, Assis, Gomes, da Silva, Fonsêca and Druzian [21], where two peaks were observed in the DTG curves within a temperature range of 200~430 °C for corn stover. It was postulated that the first peak might correspond to the decomposition of hemicellulose, while the second peak corresponded to volatile compound loss. Major constituents of lignocellulosic materials, including hemicellulose, cellulose and lignin, were reported to decompose within the temperature range of 150~500 °C [49]. Specifically, hemicellulose degraded mainly within 150~350 °C, cellulose within 275~350 °C and lignin gradually decomposed within 250~500 °C. According to Table S2 (Supplementary Materials), the residual weight of TEMPO-CNF (25.38%) is larger than that of other samples (16.66%~21.13%). This is similar to the findings reported by Jiang, Kondo and Hsieh [34] that TEMPO-oxidized rice straw cellulose (16.9% char) had a much higher residual weight than that of pure rice straw cellulose (3.3% char).

### 3.4. Thermodynamic Stability of Oil-in-Water Emulsions Stabilized by Tween 80 and TEMPO-CNF

The thermodynamic stability of oil-in-water emulsions stabilized by Tween 80 with or without the presence of TEMPO-CNF was reflected by the phase behavior of emulsions against several stability tests. As shown in Table 2, all the formulated emulsions were stable under room temperature storage for at least 30 days. The emulsions formulated with an oil/tween ratio of 3:1 formed phase separation after centrifugation with or without the presence of TEMPO-CNF, while emulsions formulated with an oil/tween ratio of 1:1 and 1:3 did not form phase separation after centrifugation. This indicates that the higher concentrations of tween 80 (i.e., lower ratio of oil/tween) present in the emulsion, the better emulsion stability against centrifugation. Tween 80 has a double bond in its hydrophobic tail, which can participate in π–π interactions, increasing the surface film rigidity and resulting in a well-defined phase boundary [50]. Higher concentrations of tween 80 could result in close packing of interfacial film, increased surface viscosity and elasticity, and, therefore, better resistance against rupture [51]. Specifically, with higher concentrations of tween 80, the percentage of oil separated during centrifugation was much lower, representing higher stability against centrifugation [51]. Under the tested experimental conditions in this study, the presence of 0.3 wt% TEMPO-CNF did not influence the centrifugal stability of Tween 80 stabilized emulsions.

As shown in Table 2, emulsions stabilized by Tween 80 only (i.e., 0% TEMPO-CNF) were not stable against two freeze–thaw cycles at oil to tween 80 ratios of 3:1 and 1:1. However, with the presence of 0.3% TEMPO-CNF, all the emulsions formulated with oil to tween 80 ratios of 3:1 and 1:1 were stable against two freeze–thaw cycles. This indicated that TEMPO-CNF enhanced the freeze–thaw stability of Tween 80 stabilized emulsions. The mechanism for TEMPO-CNF to enhance the freeze–thaw stability of Tween 80 stabilized emulsions was postulated as below: (1) both TEMPO-CNF and Tween 80 adsorbed on the emulsion oil-water interface and TEMPO-CNF formed thick interfacial membranes around emulsion droplets which can better protect them from ice crystal penetration and partial coalescence during freeze–thaw process; (2) TEMPO-CNF possessed ice recrystallization inhibition activity which could reduce the amount of frozen water and decrease the collision frequency of droplets thus improving the emulsion’s freeze–thaw stability [23].

When formulated with an oil/tween ratio of 1:1 (or 1:3) and TEMPO-CNF concentration of 0.3 wt%, the emulsions were stable against centrifugal forces, freeze–thaw cycles, and at least 30 days of room temperature storage. Therefore, the emulsions with good stability in this study include the formulations of (1) 2.5~20 wt% EO, oil/tween 80 ratios of 1:1, TEMPO-CNF 0.3 wt% and (2) 2.5~5 wt% EO, oil/tween 80 ratios of 1:3, TEMPO-CNF 0.3 wt%. One optimal formulation (i.e., 2.5 wt% EO, 7.5 wt% Tween 80 and 0.3 wt% TEMPO-CNF) was selected for further characterization and investigated for its antifungal activity.

### 3.5. Characterization of Emulsions

**Figure 4:**
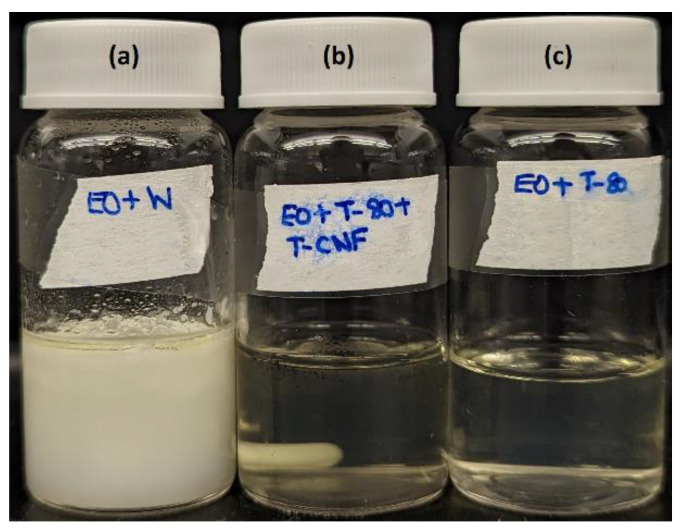
Macroscopic image of lemongrass essential oil-loaded emulsions right after preparation. (**a**) EO + W; (**b**) EO + T80 + TCNF; (**c**) EO + T80.

Table 3 shows that without the addition of Tween 80 or TEMPO-CNF, the mixture of EO and water after sonication (EO + W) had a pretty large mean particle size (619 nm) and large polydispersity index. This sample was also not visually stable over time and quickly formed phase separation. With the presence of Tween 80, the mean particle size of the emulsion (EO + T80 or EO + T80 + TCNF) reduced to 19 nm and the polydispersity index decreased as well. The mean particle size of emulsions corresponded well to their macroscopic images, as shown in Figure 4. Specifically, the sample EO + W (right after preparation) showed a milky white color due to the scattering of light by the large size droplets, while the emulsion EO + T80 or EO + T80 + TCNF showed high transparency due to its small droplet size. The generation of EO-loaded nanoemulsion stabilized by Tween 80 and/or biopolymers using ultrasonication has also been reported by others [52,53]. For instance, lemongrass essential oil-loaded alginate/Tween 80 nanoemulsion had a mean particle size of 4.3 nm [52].

The presence of TEMPO-CNF also rendered the Tween 80 stabilized emulsion more colloidally stable, with a zeta potential of −34 mV for the EO + T80 + TCNF sample as compared to that of −21 mV for the EO + T80 sample. The zeta potential of emulsions can be related to their short- and long-term stability [54]. Samples with an absolute zeta potential of larger than 30 mV are generally considered stable. In this study, Tween 80 is a nonionic surfactant while TEMPO-CNF carries a negative surface charge with a zeta potential of −65 mV; therefore, there did not exist repulsion between TEMPO-CNF and Tween 80. Instead, according to the thermodynamic stability tests in Table 2 and our previous study [23], there existed a synergistic interaction between TEMPO-CNF and Tween 80 in stabilizing oil-in-water emulsions. The surface charge of colloidal nanoparticles influenced their interaction with surfactants, which further impacted the stability of emulsions [55].

### 3.6. Antifungal Activity of Pure and Encapsulated Lemongrass Essential Oil

**Figure 5:**
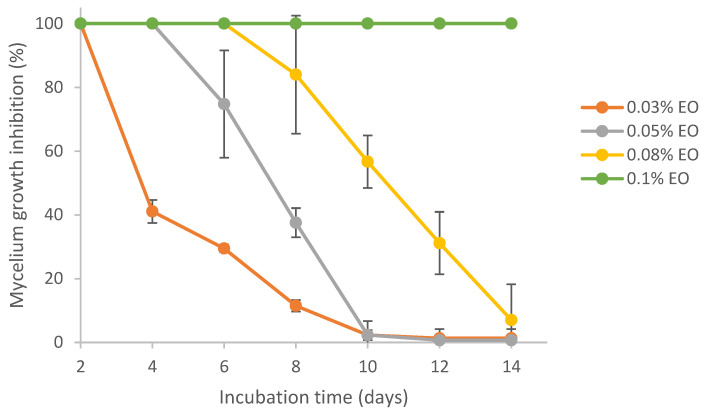
Effect of pure lemongrass essential oil on mycelial growth inhibition of *Aspergillus flavus*.

Results from Figure 5 show that lemongrass essential oil could effectively inhibit the mycelial growth of *Aspergillus flavus*. Complete colony growth (~8 cm) on the plate was observed for the control (0% EO) by day 8 (result not shown). In comparison, treatment at 0.03%, 0.05%, 0.08% and 0.1% EO resulted in 11%, 38%, 84% and 100% inhibition of mycelium growth at day 8, respectively. On day 14, the mycelial growth inhibition (MGI) rate was reduced to 7% in the presence of 0.08% EO. Lemongrass essential oil at 0.1% exhibited 100% inhibition against *A. flavus* growth for 14 days. Similarly, the fungicidal and anti-aflatoxigenic effects of lemongrass essential oil against *A. flavus* were also reported by others [56,57]. Specifically, the essential oil of *Cymbopogon citratus* (DC.) Stapf. (lemongrass) was fungistatic and fungicidal against *A. flavus* at concentrations of 0.6 mg/mL and 1.0 mg/mL, respectively. In addition, lemongrass essential oil was also reported to be one of the top three in the highest antifungal activity among 11 essential oils [58].

It was reported that the main components of lemongrass essential oil include α-citral, β-citral, d-limonene and geraniol [59]. On average, the composition of citral in lemongrass essential oil is 65~80% [60]. Citral was identified as the fungicidal constituent in lemongrass essential oil [56]. The antifungal effects of citral against *Aspergillus flavus* and *Aspergillus ochraceus* [61], as well as other fungal species [62], have also been reported. The mechanisms of antimicrobial activity of EOs generally include the interaction between phenolic compounds of EO and proteins in the cytoplasmic membrane, which causes cytomembrane disruption, as well as leakage of proteins, ions and other intracellular substances, finally resulting in cell breakdown [63,64].

**Figure 6:**
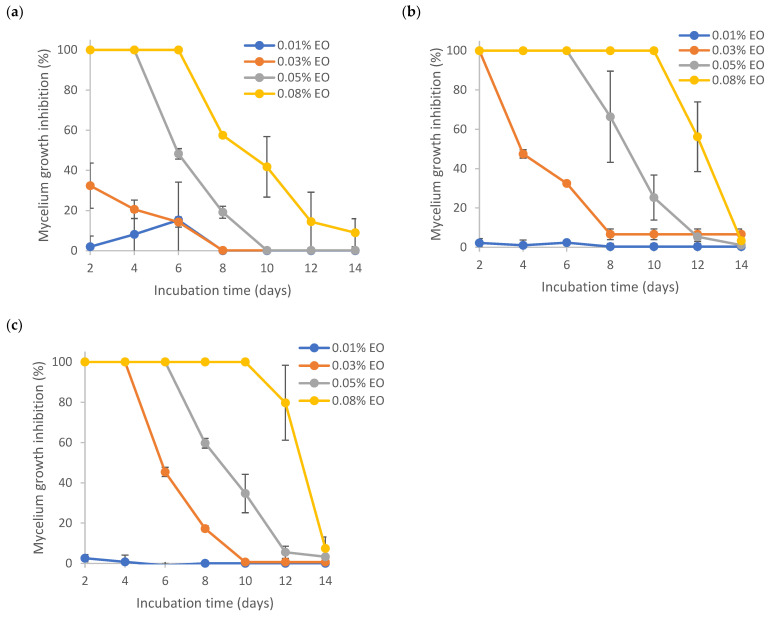
Effect of encapsulated lemongrass essential oil on mycelium growth inhibition of *Aspergillus flavus.* (**a**) EO + W; (**b**) EO + T80; (**c**) EO + T80 + TCNF.

Results from Figure 6 show the MGI rates of several encapsulated EOs. Even though all the formulations were prepared via sonication, the presence of Tween 80 or TEMPO-CNF in the formulation did make a difference in the MGI rates. By comparing the mycelial growth inhibition results of pure (Figure 5) and encapsulated (Figure 6) lemongrass essential oil at the same EO loadings (0.03%. 0.05% and 0.08% EO), it is found that the formulation EO + T80 + TCNF had the highest MGI rates, followed by the formulation EO + T80, both of which were significantly higher than that of pure EO and EO + W. No significant differences were found between the formulation EO + T80 + TCNF and EO + T80, indicating that TEMPO-CNF did not show any significant antifungal activity. This was also supported by the phenomenon that TEMPO-CNF itself (without EO) did not show any significant inhibition on mycelium growth of *Aspergillus flavus* (results not shown). The formulation EO + W had the lowest MGI, which was significantly lower than that of pure EO, indicating that EO had low solubility in water and the sonication process involved was not effective at enhancing its solubility in water or its stability. This corresponds to the description, as shown in Section 3.5, that the sample EO + W quickly separated into layers after sonication. For pure EO, its solubility in PDA media (containing 0.1% Tween 80) is higher than EO in water, though the latter had the aid of sonication. Figure S1 (Supplementary Materials) also demonstrated that no significant differences in MGI rates existed among several emulsion preparation treatments (i.e., stirring, sonication, homogenization and homogenization followed by sonication).

Figure 6 demonstrates that the nanoencapsulated lemongrass essential oil (with formulation EO + T80 + TCNF or EO + T80) had a better inhibition effect against mycelium growth of *Aspergillus flavus* than pure EO and instable microencapsulated EO (i.e., formulation EO + W). Similarly, Kapustová et al. [65] showed that nanoencapsulated thyme or oregano essential oil had better inhibition against *Aspergillus flavus* and other fungal strains as compared to pure essential oils. Specifically, the minimum fungicidal concentration (MFC) of nanoencapsulated thyme essential oil against *Aspergillus flavus* was 0.5 mg/mL, while the MFC of pure thyme essential oil was 1 mg/mL. Superior bactericidal activity of nanoemulsion over coarse emulsions has also been reported for lemongrass essential oil-loaded nanoemulsion [66]. Lavender oil-loaded nanoemulsion was also found to have a better inhibitory effect against fungal strains such as *A. flavus* than pure oil [67].

Encapsulation of essential oils in nano-size was shown to increase its antimicrobial activity. For instance, Donsì et al. [68] showed that nanoencapsulation of tea tree EO was more effective at inhibiting the growth of three types of microorganisms (*Saccharomyces cerevisiae*, *Escherichia coli* and *Lactobacillus delbrueckii*) as compared to non-encapsulated EO. The antimicrobial efficacy of the nanoencapsulated EO depended on the mean size and formulation of the delivery system as well as the microorganism [68]. Liolios et al. [69] also found that encapsulated EO had higher antimicrobial effects than non-encapsulated EO.

The mechanisms of action of nanoencapsulated essential oil against microorganisms were described in the literature [70]. Specifically, the nano-size of nanoencapsulated EO has a larger specific surface area; thus, the interaction between EO and microorganism cell membrane was more efficient. In addition, the encapsulation material can enhance the stability and dispersibility of EO and deliver EO to specific target sites. EO can be transported across the cell membrane and released inside the cytoplasmic membrane [70].

**Figure 7:**
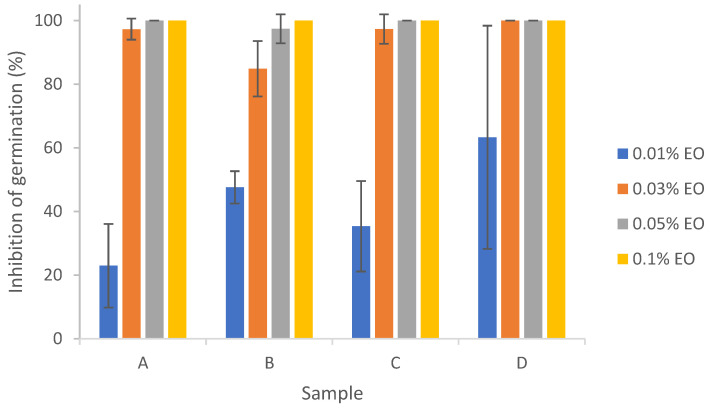
Effect of pure and encapsulated lemongrass essential oil on inhibition of germination of *Aspergillus flavus.* (A) EO + W; (B) EO + T80; (C) EO + T80 + TCNF; (D) pure EO.

Results from Figure 7 show that 0.03%~0.1% lemongrass essential oil with or without encapsulation was effective in inhibiting the germination of *Aspergillus flavus*, with percent inhibition of germination (IG%) around 100%. Significant differences in IG% were found between 0.01% EO and EO concentrations ≥ 0.03%. There did not exist any significant differences between pure EO (D) and encapsulated EO (A, B, or C), indicating that the emulsion stabilizers and/or sonication did not significantly impact the action of EO on inhibition of conidial germination of *Aspergillus flavus*. Antifungal effects of lemongrass essential oil on the conidial germination of other fungi (e.g., *Cladosporium herbarum*) at an EO concentration of 0.5% have been previously reported [26]. Complete inhibition of *A. flavus* germination could also be achieved by other citrus EOs like bitter orange and bergamot at 2% [26].

Combining the results of the mycelium growth assay and inhibition of germination assay (Figure 5, Figure 6 and Figure 7), nanoencapsulated essential oil exhibited enhanced inhibition against *Aspergillus flavus* as compared to pure essential oil, indicating that nanocellulose is a suitable material for lemongrass essential oil nanoencapsulation. Similarly, nanocellulose has been shown to be a suitable material support for limonene, eugenol and cinnamaldehyde [71]. Other biopolymers, such as chitosan [72] and poly(ε-caprolactone) [73], have also been shown to be suitable material supports for essential oil nanoencapsulation that enhances the antimicrobial efficacy of essential oils. The enhanced antimicrobial efficacy of essential oils by nanoencapsulation demonstrated its potential application in food and agricultural fields to reduce pathogens and plant diseases. The potent antimicrobial efficacy of essential oil as a biopesticide could also be a more natural alternative to chemical pesticides to reduce the adverse effects the latter exerts on the environment.

## 4. Conclusions

In this study, TEMPO-CNF with a crystallinity index of 69.5% was prepared from corn stover. Corn stover-derived TEMPO-CNF contained carboxyl groups according to FTIR, and it had lower thermal degradation temperature as compared to other corn stover samples. Lemongrass essential oil could be nanoencapsulated by corn stover-derived TEMPO-CNF and Tween 80 in a nanoemulsion format. The nanoemulsion stabilized by this binary emulsion stabilizer (i.e., TEMPO-CNF and Tween 80) had a mean particle size of 19 nm and a surface charge of −34 mV. The nanoemulsion formulated with TEMPO-CNF and Tween 80 was physically stable against centrifugal forces, freeze–thaw cycles and at least 30 days of room temperature storage. The antifungal activity against *Aspergillus flavus* for lemongrass essential oil-loaded nanoemulsion was higher than that of pure essential oil, indicating the effective encapsulation of lemongrass essential oil in the nanostructure. Findings from this study suggest that corn stover-derived nanocellulose is an appealing candidate for encapsulation of essential oils with enhanced antimicrobial activity and thermodynamic stability. The developed biopesticides can be an alternative to chemical pesticides and reduce the negative impact of chemical pesticides on ecosystems and human health. This complies with the increased consumer demand for safe and natural products.

## Figures and Tables

**Table 1:** Crystallinity index of corn stover samples after each treatment.

Sample	Crystallinity Index (%)
Corn stover after washing (CS)	57.07
Corn stover after bleaching treatment (CS-BT)	67.20
Corn stover after bleaching and alkaline treatment (CS-BAT)	69.95
Corn stover after TEMPO oxidation (TEMPO-CNF)	69.50

**Table 2:** Phase behavior of emulsions stabilized by Tween 80 and TEMPO-CNF against thermodynamic stability tests.

Oil/Tween Ratio	Lemongrass Essential Oil (wt%)	Tween 80 (wt%)	TEMPO-CNF (wt%)	After Centrifugation	After 2 Freeze–Thaw Cycles	After 30 Days Storage
3:1	2.5	0.83	0	PS ^1^	PS	NPS ^2^
	5	1.67	0	PS	PS	NPS
	10	3.33	0	PS	PS	NPS
	20	6.67	0	PS	PS	NPS
	2.5	0.83	0.3	PS	NPS	NPS
	5	1.67	0.3	PS	NPS	NPS
	10	3.33	0.3	PS	NPS	NPS
	20	6.67	0.3	PS	NPS	NPS
1:1	2.5	2.5	0	NPS	PS	NPS
	5	5	0	NPS	PS	NPS
	10	10	0	NPS	PS	NPS
	20	20	0	NPS	PS	NPS
	2.5	2.5	0.3	NPS	NPS	NPS
	5	5	0.3	NPS	NPS	NPS
	10	10	0.3	NPS	NPS	NPS
	20	20	0.3	NPS	NPS	NPS
1:3	2.5	7.5	0.3	NPS ^3^	NPS ^3^	NPS
	5	15	0.3	NPS ^3^	NPS ^3^	NPS

Note: ^1^ PS refers to phase separation, ^2^ NPS corresponds to no phase separation, ^3^ the result was reprinted from our previous study [23] with permission from the publisher.

**Table 3:** Particle size and zeta potential characterization of lemongrass essential oil-loaded emulsions.

Sample Label	Lemongrass Essential Oil (wt%)	Tween 80 (wt%)	TEMPO-CNF (wt%)	Mean Particle Size (nm)	Polydispersity Index	Zeta Potential (mV)
EO + W	2.5	0	0	619 ± 86	0.69 ± 0.09	−53 ± 4
EO + T80	2.5	7.5	0	19 ± 2	0.46 ± 0.06	−21 ± 9
EO + T80 + TCNF	2.5	7.5	0.3	19 ± 2	0.63 ± 0.08	−34 ± 6

## Data Availability

The data presented in this study are available on request from the corresponding author.

## Supplementary Materials

The following supporting information can be downloaded at: https://www.mdpi.com/article/10.3390/polym15193946/s1, Figure S1: Effect of different emulsion preparation treatments for encapsulated lemongrass essential oil on the mycelium growth inhibition against *Aspergillus flavus*. (a) stirring; (b) sonication; (c) homogenization; (d) homogenization followed by sonication. The emulsion was formulated with 2.5% lemongrass essential oil (EO), 7.5% Tween 80 and 0.3% TEMPO-CNF.
