# Supplementary material for: Characterization and Antifungal Activity of Lemongrass Essential Oil-Loaded Nanoemulsion Stabilized by Carboxylated Cellulose Nanofibrils and Surfactant
Source: Polymers (Basel). 2023 Sep 29;15(19):3946. doi: 10.3390/polym15193946 (PMC10575251; doi:10.3390/polym15193946)
Supplement: Supplementary file 1 [file polymers-15-03946-s001.zip › polymers-2579717-supplementary.pdf]

Supplementary Material

Characterization and antifungal activity of lemongrass essential oil-loaded nanoemulsion stabilized by carboxylated cellulose nanofibrils and surfactant

Lingling Liu [a,*], Kaleb D Fisher [b], Mason A Friest [c], Gina Gerard [d]

[a]*Department of Agricultural and Biosystems Engineering, Iowa State University, Ames, IA 50010, USA*

[b]*Roy J. Carver Department of Biochemistry, Biophysics and Molecular Biology, Iowa State University, Ames, IA 50010, USA*

[c]*Department of Mechanical Engineering, Iowa State University, Ames, IA 50010, USA* (mafriest@iastate.edu)

[d]*Department of Food Science and Human Nutrition, Iowa State University, Ames, IA 50010, USA*

*** Correspondence:** lingling@iastate.edu

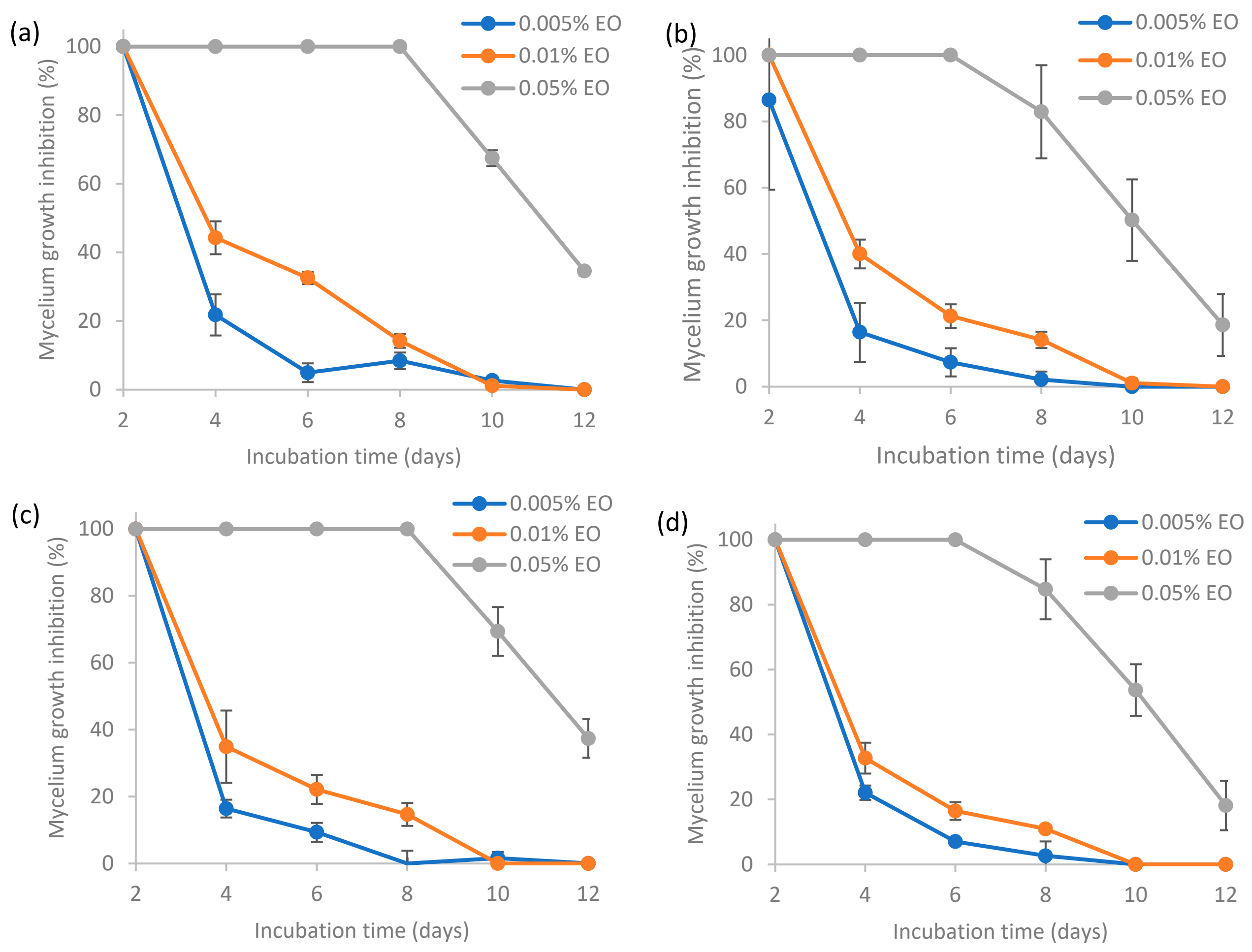

Figure S1. Effect of different emulsion preparation treatments for encapsulated lemongrass essential oil on the mycelium growth inhibition against *Aspergillus flavus*. (a) stirring; (b) sonication; (c) homogenization; (d) homogenization followed by sonication. The emulsion was formulated with 2.5% lemongrass essential oil (EO), 7.5% Tween 80 and 0.3% TEMPO-CNF.
